# Perception of rigidity in three- and four-dimensional spaces

**DOI:** 10.3389/fpsyg.2023.1180561

**Published:** 2023-08-17

**Authors:** Dongcheng He, Dat-Thanh Nguyen, Haluk Ogmen, Shigeaki Nishina, Arash Yazdanbakhsh

**Affiliations:** ^1^Department of Electrical and Computer Engineering, Laboratory of Perceptual and Cognitive Dynamics, University of Denver, Denver, CO, United States; ^2^Herbert Wertheim School of Optometry and Vision Science, University of California, Berkeley, Berkeley, CA, United States; ^3^Department of Psychological and Brain Sciences, Computational Neuroscience and Vision Lab, Center for Systems Neuroscience, Graduate Program for Neuroscience, Boston University, Boston, MA, United States; ^4^Honda Research Institute Japan Co., Ltd., Wako, Japan

**Keywords:** hyperspace perception, 3D motion perception, rigid motion, virtual reality, spatial cognition

## Abstract

Our brain employs mechanisms to adapt to changing visual conditions. In addition to natural changes in our physiology and those in the environment, our brain is also capable of adapting to “unnatural” changes, such as inverted visual-inputs generated by inverting prisms. In this study, we examined the brain’s capability to adapt to hyperspaces. We generated four spatial-dimensional stimuli in virtual reality and tested the ability to distinguish between rigid and non-rigid motion. We found that observers are able to differentiate rigid and non-rigid motion of hypercubes (4D) with a performance comparable to that obtained using cubes (3D). Moreover, observers’ performance improved when they were provided with more immersive 3D experience but remained robust against increasing shape variations. At this juncture, we characterize our findings as “3 1/2 D perception” since, while we show the ability to extract and use 4D information, we do not have yet evidence of a complete phenomenal 4D experience.

## 1. Introduction

Visual information about our environment is projected as two-dimensional images on our retinae. By using various cues, such as disparity, motion parallax, perspective, and shading, the visual system *constructs* three-dimensional percepts from these two-dimensional projections. For example, due to the lateral separation between two eyes, a point in the external environment is projected onto different locations between two retinae, and this positional difference is called “binocular disparity” and is well known to contribute to the depth perception of our visual system. Another source of information being used to produce depth perception is motion parallax: a nearer point’s retinal projection moves faster than a distant one, and vice versa, when they move by the same velocity or when the fixation point moves. In addition to these oculomotor cues, pictorial cues, such as shading and perspective, also play an important role in depth perception, especially in monocular vision. The role of these cues in three-dimensional perception has been investigated and explained in psychophysical studies and related neural models are suggested (Gibson et al., 1959; Rogers and Graham, 1979; Cormack and Fox, 1985; Bülthoff and Mallot, 1988; Biederman and Gerhardstein, 1993; Qian, 1997; Langer and Bülthoff, 2000; Read, 2005; Saxena et al., 2008; He and Ogmen, 2021). Altogether, the human brain is capable of augmenting the dimensionality of incoming visual inputs.

A question arises as to whether our brains are limited to three-dimensional representations. On the one hand, one may argue that, since we live in a three-dimensional environment, our brains are evolved to represent three-dimensional percepts only. On the other hand, our brains show *plasticity*, i.e., the capability of adapting to changing conditions, such as experience during brain development, brain injury, and aging effects (Aoki and Siekevitz, 1988; Wieloch and Nikolich, 2006; Brehmer et al., 2014). Vision does not start in a mature state in infants; rather it is through visual adaptation and developmental processes that it reaches its mature state (Riesen, 1947; Kellman and Arterberry, 2007; Johnson, 2011). Visual development requires interactions with the environment. The plasticity associated with these interactions is not limited to infants but also continues in adulthood. For example, using inverting lenses, Stratton (1896) showed that we can adapt to image inversion and carry out complex tasks successfully, such as riding a bicycle in city streets. Given this ability to adapt to “unnatural conditions,” one may postulate that, with sufficient experience with higher-dimensional (four or more spatial dimensions) stimuli, our brains may be able to adapt to these higher spatial-dimensional stimuli. This hypothesis can be tested experimentally. Even though our environment is three-dimensional, mathematically we can generate higher-dimensional stimuli (“hyperspaces”) and expose subjects to these stimuli.

In fact, previous studies have shown that human observers can perform better than chance in hyperspace reasoning or hyperdimensional object recognition tasks (review: Ogmen et al., 2020). In these studies, along with the presentation of 4D stimuli, tasks had different dimensional levels. In 1D and 2D tasks, geometrical concepts like distance and angle were used and it was found that observers can measure approximately distances and angles within 4D tetrahedrons (Ambinder et al., 2009). Wang (2014a,b), investigated if subjects were able to measure the hypervolume of 4D objects by learning rotated 4D objects and found that subjects showed the ability to judge the actual hypervolume.

Experiments with higher-than-3D level tasks can be more difficult due to the complexity of information provided and the technical limits of devices to present the stimuli. In Aflalo and Graziano’s (2008) study, subjects were found to effectively learn to navigate in 4D mazes constructed from 2D displays. To construct and present a 4D object in the 2D display, this study used different colors to represent different axes so that planes defined by axis pairs can be differentiated on a 2D screen even though these planes can be perpendicular to each other. Experiments using a single 2D display might suffer information loss and distortion since depth on a 3D level along two directions can be confounded with each other. The use of Virtual Reality could ameliorate this limitation by providing a more immersive display environment and allow more interactive tasks. For instance, Miwa et al. (2018) in their study presented different perspectives of the 3D projections of the 4D objects and let the subjects actively interact with the presentation by manually controlling the rotation of objects via a VR system. Performance suggested that subjects were able to reconstruct the 4D space based on multiple 3D projections.

As shown above, most of the previous studies investigated hyperspace perception by designing stimuli and tasks related to spatial reasoning, spatial relations, and perspective learning. We focused not on spatial perception but rather on shape perception capabilities, investigating whether it is possible to reconstruct four-dimensional structures of objects moving in the four-dimensional space from the visual inputs. Motion is a key source of information in visual perception. Among the diverse cues deployed for perceiving three-dimensional structures based on retinal images, motion parallax plays a major role in perceiving the third dimension from two-dimensional stimuli (Gibson et al., 1959; Rogers and Graham, 1979). Whether and how motion can be used to generate higher-dimensional percepts remains largely unexplored. Here we focused on the perception of rigidity. The rigidity of a shape serves as a critical cue for recognizing the identity of a shape as the perspective changes (Gibson and Gibson, 1957; Ullman, 1984). We are often able to identify stable objects across transformations and have strong subjective impressions of the transformations themselves (Boring, 1940; Rock et al., 1968; Pizlo, 1994). This suggests that the brain is equipped with sophisticated mechanisms for inferring both object constancy and objects’ causal history. The ability to perceive and classify the rigidity of object transformation is thus an important aspect of spatial representations. During the process of a three-dimensional shape being projected onto the retina, a dimension is lost. Consequently, even if the original shape was rigid in its original dimensions, the projected two-dimensional image does not maintain this rigidity. Despite this, we are able to judge the rigidity of the original object. This can be accomplished only by reconstructing the shape in its original dimensions from the retinal image. By focusing on the performance in perceiving rigidity, it becomes possible to investigate the observer’s ability to reconstruct higher-dimensional shapes.

In this study, we used a VR system to present subjects with cubes (3D) and hypercubes (4D) undergoing either rigid or non-rigid motion and tested their ability to judge the rigidity of the stimuli. The cubes and hypercubes were either regular (with orthogonal sides and surfaces) or having various levels of deviation from regular configuration. The observers’ reaction time, hit rate, and confidence in the judgment were recorded to measure their performance on the basis of ease of detection and cognitive access. We asked subjects to report their confidence levels to help us understand if they can generate the phenomenal experience of the hypercube motion, or in other words, if they were aware of what they observed during the experiments. We hypothesized that the necessary condition for experiencing phenomenal hyperspace is that human observers’ performance in 4D motion rigidity judgment task equals their performance in 3D condition. Moreover, we varied axes of motion and object regularities factors to test the dependency of observers’ performance on each factor.

## 2. Materials and methods

### 2.1. Subjects

Six students (two women and four men; age: *M*[SD] = 21[3.16] years), including one of the authors, from the University of Denver participated in the first experiment and all participants had a normal or corrected-to-normal vision. Five subjects (one woman and four men; age: *M*[SD] = 22.3[2.62] years) who attended experiment 1 participated in the second experiment. These experiments followed a protocol approved by the University of Denver Institutional Review Board for the Protection of Human Subjects. Each observer gave written consent before the experiment.

### 2.2. Apparatus

In this experiment, we presented our stimulus using an HTC VIVE VR headset released in 2017. The VR headset possesses two rectangular screens with circular lenses for the two eyes, each with a diagonal size of 91.4 mm. The resolution of each screen was 1,200 × 1,080 with a 90 Hz refresh rate. The approximate pupil-to-lens distance was 18 mm. The display of the device is controlled by the development environment Unity3D.

### 2.3. Description of stimuli

**Figure 1:**
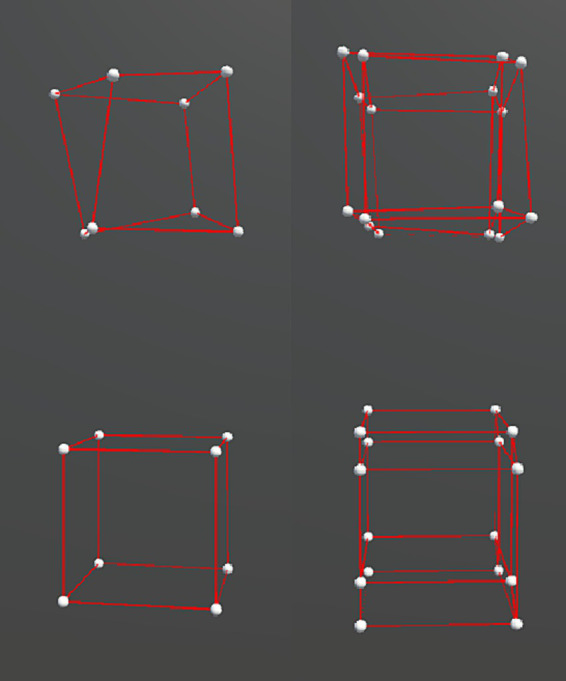
Distorted cubes used in the experiment. The left and right columns show the cubes in 3D and 4D, respectively. The bottom row contains the original cubes, and the top row contains the cubes after adding the distortion.

**Figure 2:**
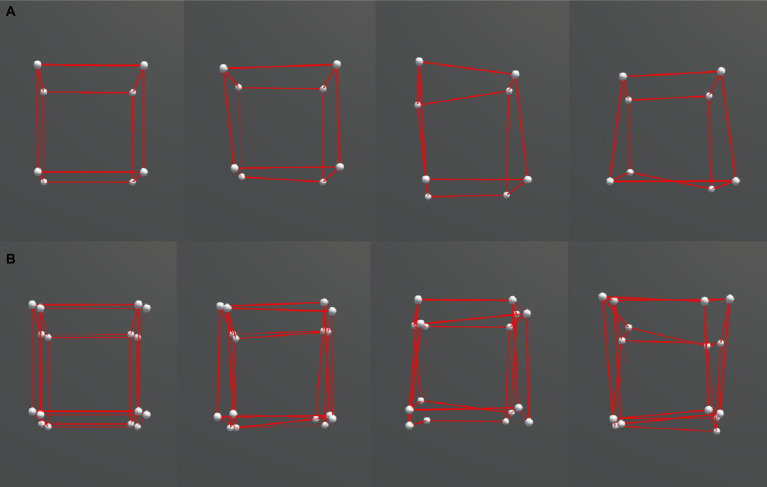
Comparison between different ranges of irregularity. From left to right: 0, 0.12, 0.18, 0.24. **(A)** 3D cubes. **(B)** 4D cubes.

The stimuli were distorted cube (3D) and hypercube (4D) wireframe objects (Figure 1). A cube consisted of 8 vertices and 12 edges, and a hypercube consisted of 16 vertices and 32 edges. The objects were presented in a 3D virtual space using the VR headset. For the hypercube, we presented its 3D projection. We made the distorted object shape by shifting the position of each vertex of a regular cube or hypercube with side lengths of 100 cm in a random direction by one of the three amounts: 12, 18, or 24 cm, representing the three irregularity levels, as shown in Figure 2. These positional shifts were added on a three-dimensional level and thus violated the interspatial relations among the vertices of a hypercube during its motion. Our pilot experiments showed that larger irregularity levels could break subjects’ perceptual experience of 4D, and interconnected 3D objects were perceived instead.

**Figure 3:**
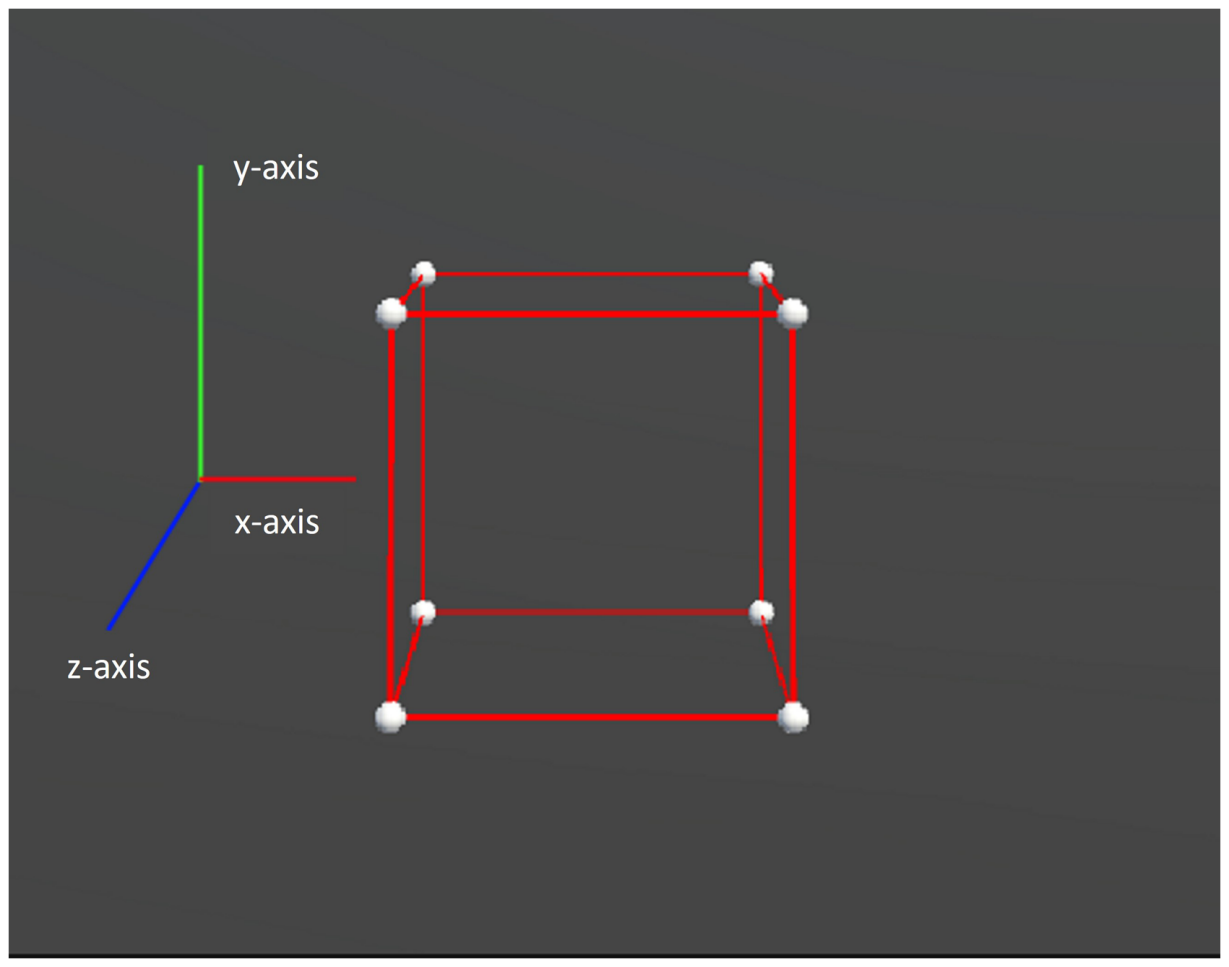
Axis legend for the virtual experiments. The x-axis represents left to right, the y-axis represents up to down, and the z-axis represents in to out.

**Figure 4:**
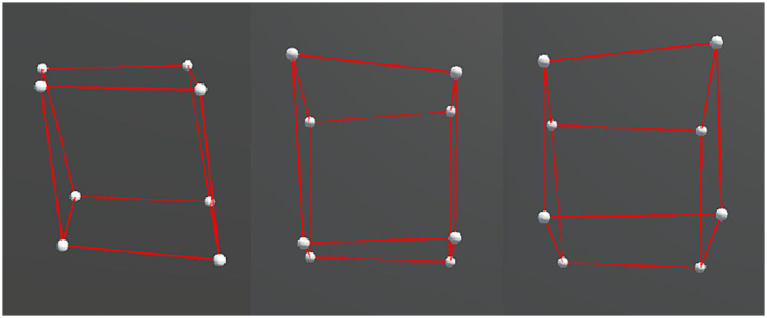
Examples of each displacement axis condition, from left to right: x-axis (left–right) displacement, z-axis (in-out) displacement, and y-axis (up-down) displacement.

We presented two types of object motion in the experiment: rigid and non-rigid. For both types, a slight random motion was applied to each vertex to enhance the stereoscopy of the object. Specifically for the rigid motion, the entire object rotated repeatedly over a fixed angular range around the x and y axes, and the axial information is depicted in Figure 3. Generalized to the hypercubes, it was a plane, instead of a line, that serves as the rotation axis, and thus the hypercubes rotated along y-z, x-z, and x-y planes. Whereas for the non-rigid motion, in addition to the same rotation as the rigid motion, non-rigidity was made by simultaneously deforming the object along random directions and adding the axial displacements via shearing to the x-, y-, or z-axis, as shown in Figure 4. The mathematical descriptions of the stimuli can be found in the Supplementary material.

In Experiment 1, The distance from the subject’s eyes to the center of the object in the virtual space was fixed. The objects were presented in perspective projection. In addition to the perspective cue, depth information in the 3D presentation was collaboratively given by (1) a binocular cue provided by the stereoscopic effect of VR and (2) a structure-from-motion cue generated by randomly rotating the entire object. Additionally, a more prominent motion parallax cue was provided in Experiment 2, by allowing the observers to move their heads actively while exposed to variable perspective levels.

### 2.4. Experimental design

#### 2.4.1. Experiment 1

**Figure 5:**
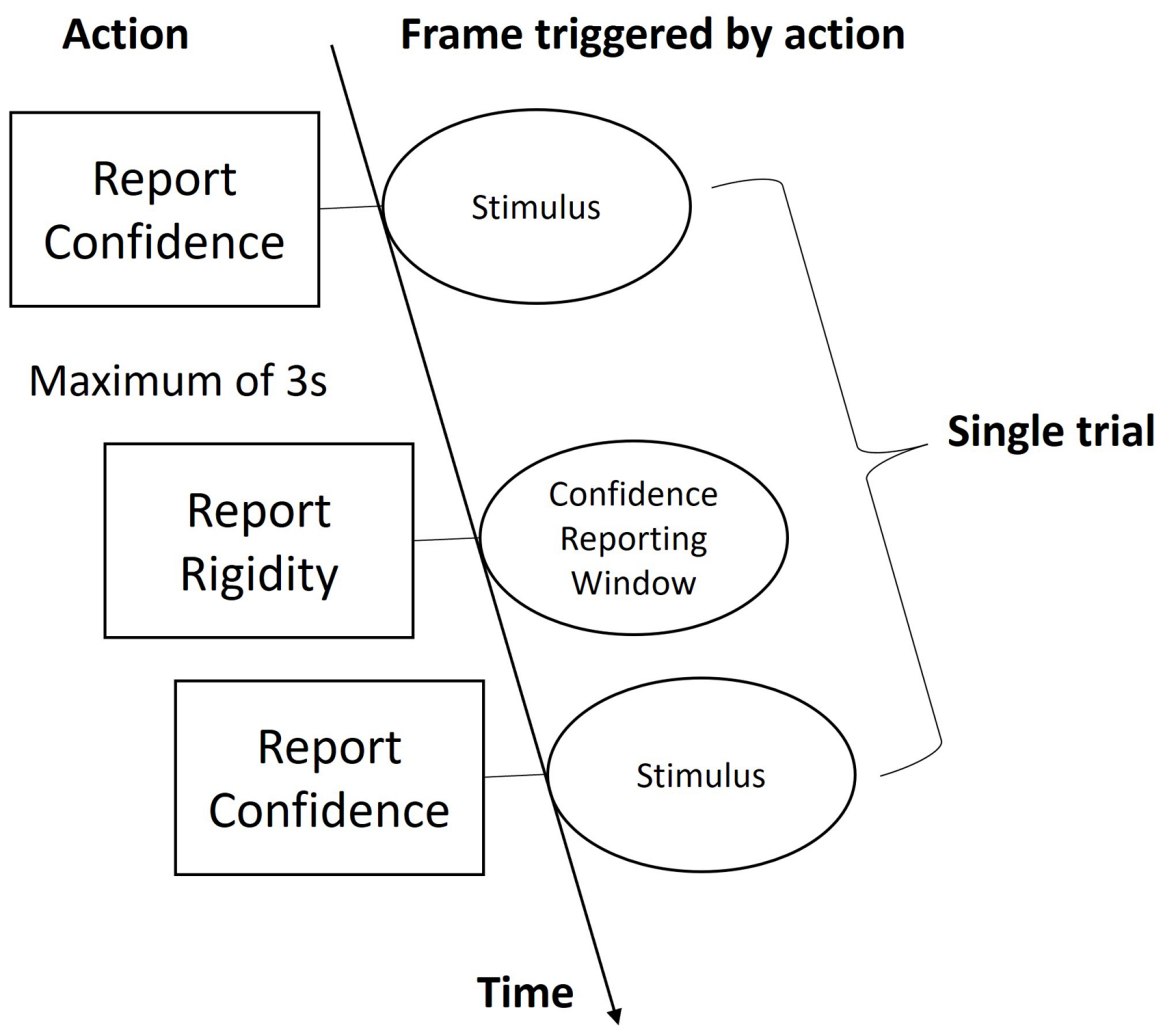
Schematic of a trial.

In a normally illuminated room, subjects sat on a chair and observed the stimuli via the VR headset that was placed on a rack fixed on the table. The viewpoint of the observation was fixed toward the front view of the stimulus regardless of the headset’s position and rotation. As shown in Figure 5, beginning of each trial, two cubes were presented in a top-bottom manner, in which one had rigid motion and the other had non-rigid motion. With text notification, subjects were guided to determine which one of the two cubes has rigid motion by pressing one of the two buttons of a mouse (press the left button to report the top and the right button to report the bottom cube). This window would last for up to 3 s. Timing-out would trigger the next trial automatically and thus overtime responses would not be recorded. Once reported, the next window would come when subjects were asked to report their confidence in the previous decision by pressing a key from 1 to 5 on a keyboard, where 1 means purely guessing and 5 means firm belief. The next trial would come as soon as they pressed a key. A video demo showing a trial can be found on the following link: https://github.com/hedch/4D_reconstruction. A session consisted of objects of two dimensionalities (3D and 4D) in motion along three displacement axes (x, y, and z), and by three irregularity levels (0.12, 0.18, and 0.24). Each case was repeated by three times, and thus there were 54 trials in a session. 3D and 4D trails were separated by text instruction and together with a time interval of 5 s. Within each dimensional condition, the sequence of presentations was randomized in a counter-balanced order. Each subject took three sessions of experiments, in which the first session was for training purposes and thus wasn’t included in the analysis.

#### 2.4.2. Experiment 2

The single trial procedure was the same as in Experiment 1. However, in this experiment, the x-axis displacement was removed. Therefore, a session consisted of objects of two dimensionalities (3D and 4D) in motion along two displacement axes (y and z), and by three irregularity levels (0.12, 0.18, and 0.24). Each case was repeated by seven times, and thus there were 84 trials in a session. Due to the previous experience of all subjects, no training session was done, and each subject was asked to finish two sessions of experiments with about ten minutes of resting between them. Importantly, observers wore the headset during the experiment and their viewpoint in the VR environment was changed accordingly as they move or rotate their heads. All other experimental settings were the same as Experiment 1.

### 2.5. Results

In the following analyses, we removed all the trials with overtime responses, which account for 63 out of 1,380 trials. Also, for the analysis based on reaction times, only trials with correct answers were included.

#### 2.5.1. Experiment 1

Among the six subjects, one had a performance of 54.9% and a reported averaged confidence of 1.71 in the experiments and whose data were therefore excluded from the analysis. The percentages of the correctness (averaged confidence) of the other five subjects are 79.21% (4.3), 69.66% (3.1), 82.52% (4.44), 79.8% (4.47), and 65.09% (4.08) respectively.

**Figure 6:**
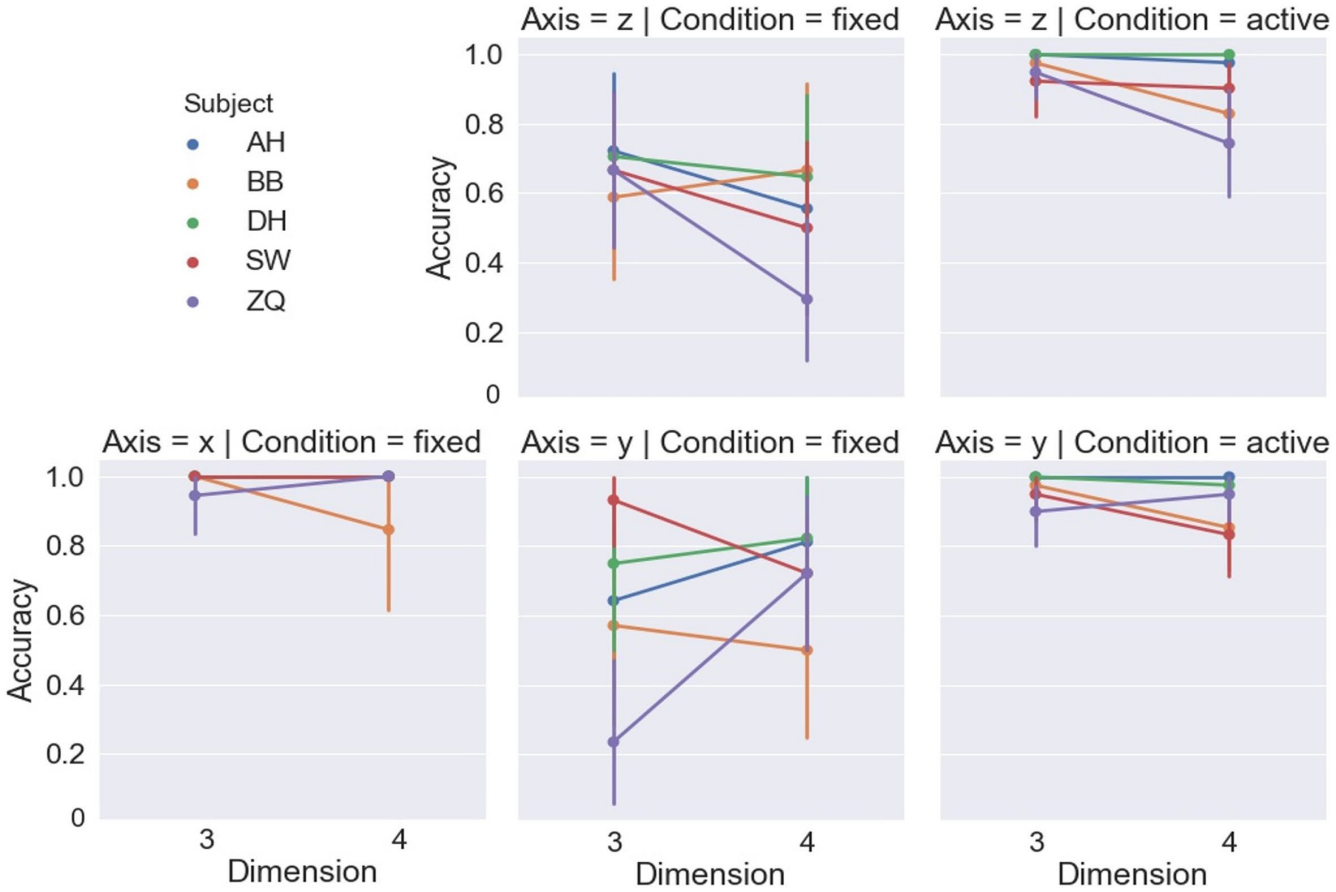
Accuracy of each subject in each condition. All error bars from Figures 7–11 are drawn with 95% confidence intervals.

Figure 6 shows accuracy in terms of percent-correct responses for different stimulus dimensions and displacement axes. For the fixed-headset condition, a linear mixed-effect model based ANOVA (LMM-ANOVA), with displacement axes and dimensions as fixed effect variables and with subjects as the random effects grouping factor, showed a significant effect of displacement axis [*F*(2,5.15) = 31.14, *p* = 0.001] on accuracy. Specifically, as detailed in Table 1, the performance under the x-displacement condition was remarkably better than the other two displacement axes. The effect of dimension was found to be non-significant on accuracy [*F*(1,6.58) = 0.29, *p* = 0.61], and there was no significant interaction between displacement axes and dimension [*F*(2, 5.2) = 0.81, *p* = 0.49]. One subject (ZQ) showed lower than chance level accuracies in two conditions: y-axis, 3D and z-axis, 4D, suggesting a strong confusion between rigid and non-rigid motion when observed presentations in such conditions. While his performance in the x-axis condition in the same experiment indicated no conceptual misunderstanding between the terms of rigidity and rigid motion, these results suggest strong difficulty in detectability of rigid motion instead of just differentiation between rigid and non-rigid motions.

**Table 1:** Fixed headset condition: 3D measurement, 4D measurement in means (std).

	x-axis displacement	z-axis displacement	y-axis displacement
Accuracy (%)	98.84 (10.78), 97.59 (15.43)	67.05 (47.27), 52.5 (50.25)	61.84 (48.9), 71.76 (45.28)
Reaction Time (ms)	580.19 (317.86), 529.45 (256.19)	782.63 (343.97), 1019.79 (429.32)	847.7 (354.2), 726.29 (389.6)
Confidence	4.94 (0.32), 4.67 (0.87)	3.73 (1.35), 3.35 (1.31)	4.03 (1.12), 3.86 (1.18)

**Figure 7:**
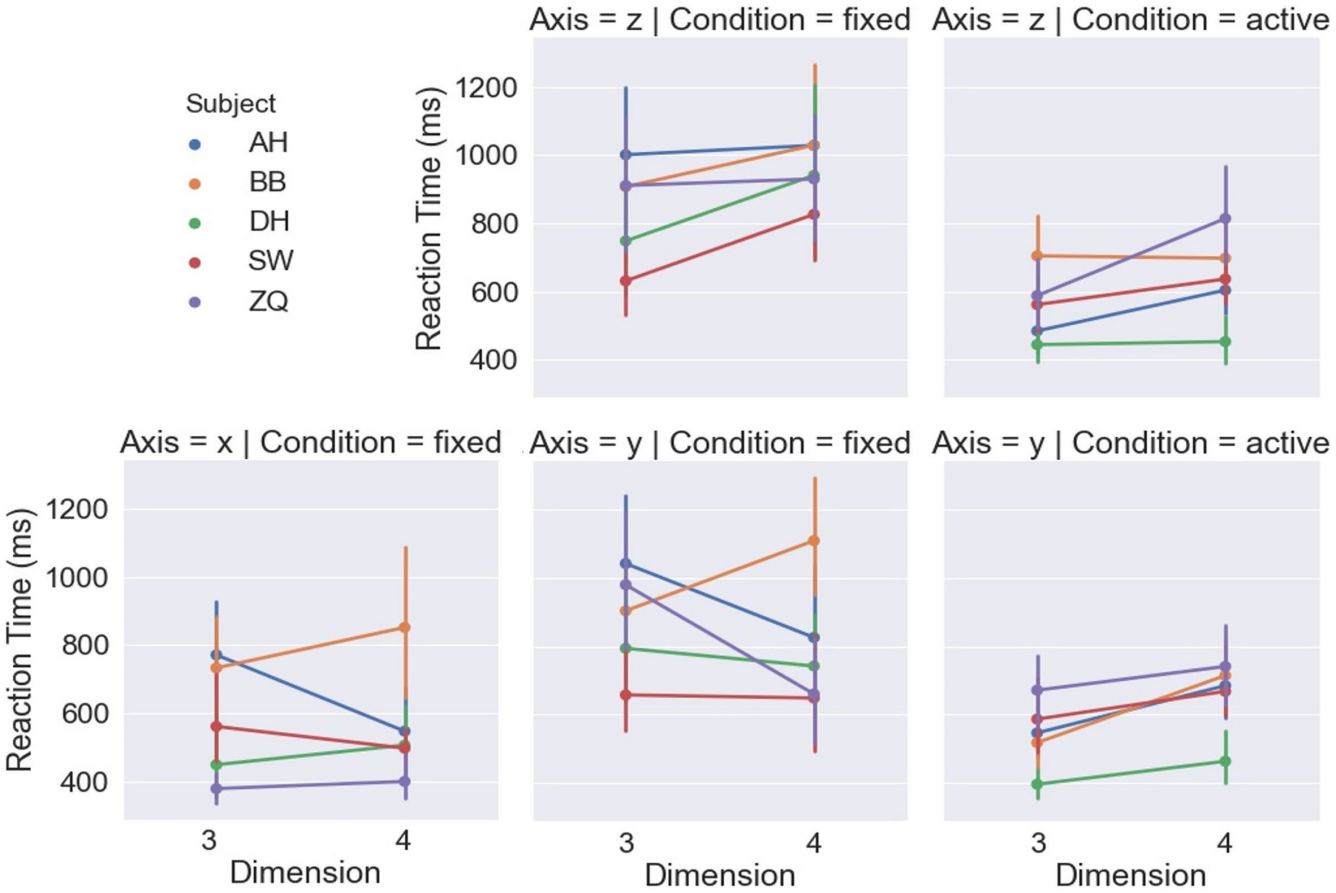
Reaction times of each subject in each condition.

Figure 7 shows the reaction time by the dimensions and displacement axes. For the fixed-headset condition, a LMM-ANOVA showed a significant effect of displacement axes [*F*(2,5.35) = 13.41, *p* < 0.01] on reaction time. Coinciding with the accuracy, a shorter time was spent in the rigidity discrimination task when the vertices were displaced along the x-axis compared to the other two axes, as shown in Table 1. The effect of dimension was found to be non-significant on reaction time [*F*(1,3.714) = 0.1, *p* = 0.77]. However, the interaction between the displacement axis and dimension was found to be significant [*F*(2,6.25) = 9.29, *p* = 0.01], which can be interpreted by the inverse effects of dimension under the y-axis and z-axis displacements.

**Figure 8:**
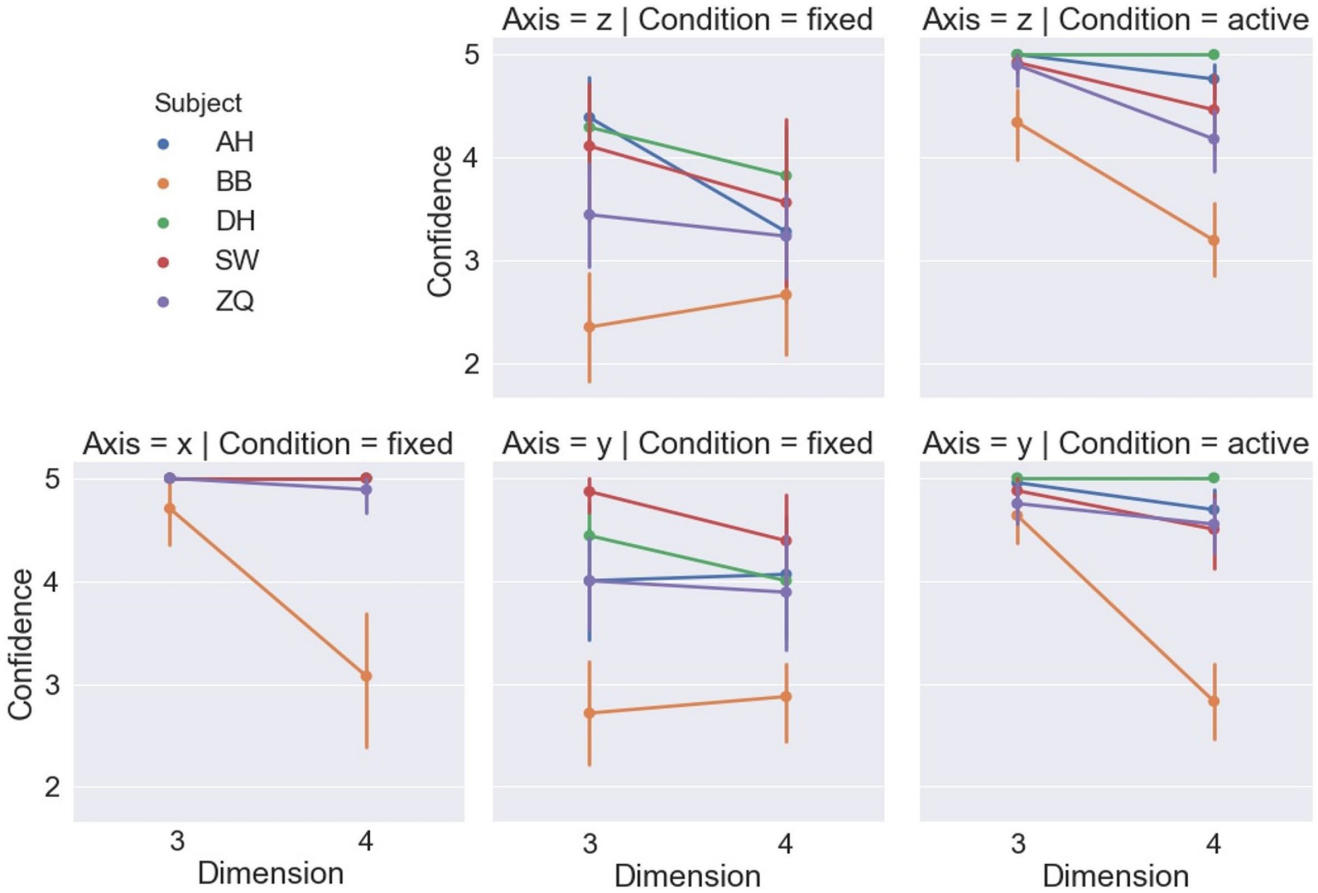
Confidence levels of each subject in each condition.

Subjects’ reported confidence levels are shown in Figure 8, and a LMM-ANOVA showed a significant effect of the displacement axis [*F*(2,4.72) = 21.44, *p* < 0.01] on the confidence level. In general, subjects were more confident when the cubes m along the x-axis in contrast to the other two axes, which was consistent with their behaviors reflected by the reaction time and accuracy. Also, dimension was found to have no significant effect on confidence level [*F*(1,4.92) = 2.06, *p* = 0.21], and no significant interaction between displacement axis and dimension was detected [*F*(2,5.32) = 4.76, *p* = 0.07].

**Figure 9:**
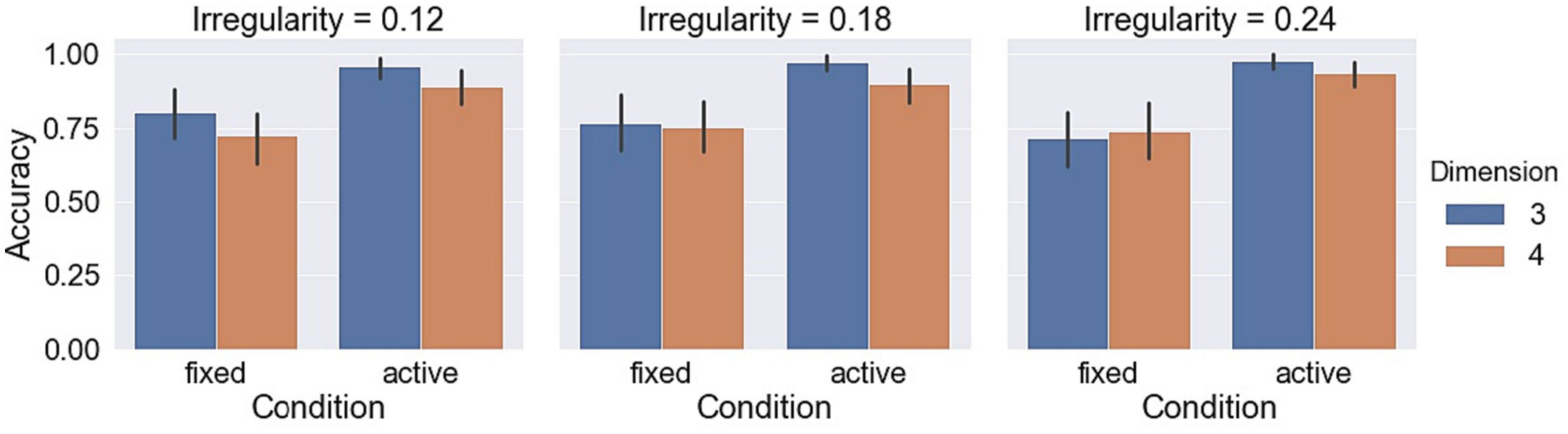
Accuracy in different object irregularity conditions.

**Figure 10:**
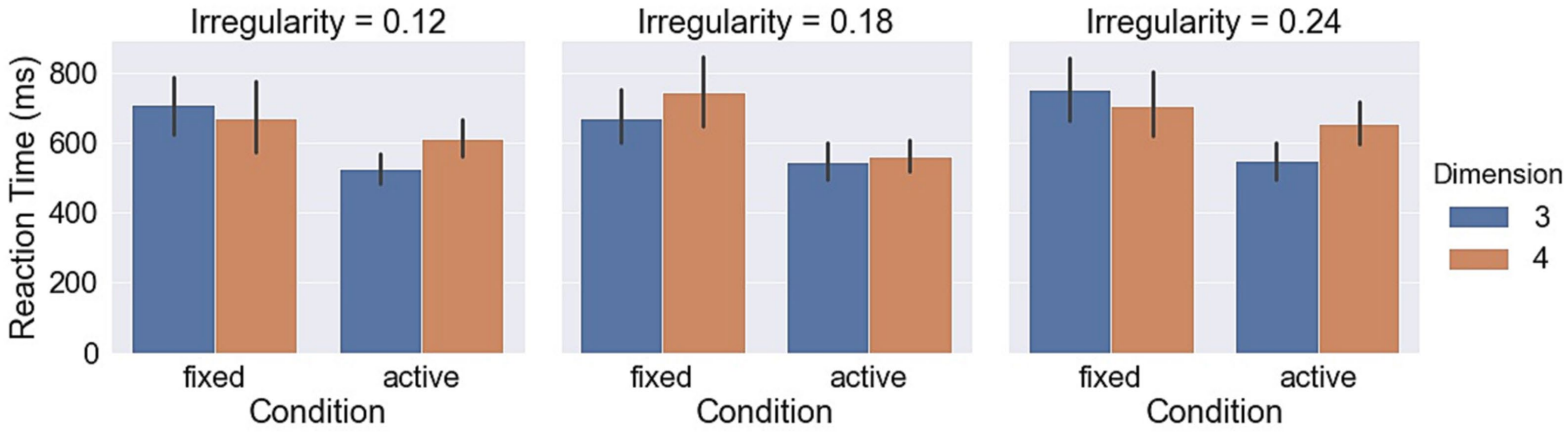
Reaction times in different object irregularity conditions.

**Figure 11:**
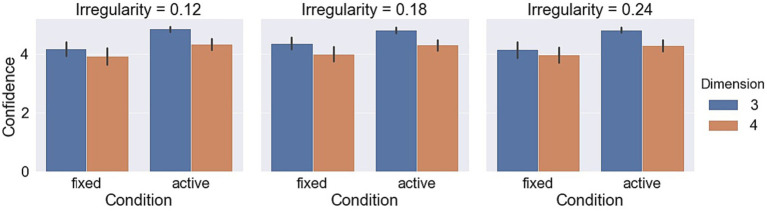
Confidence levels in different object irregularity conditions.

Measurements with respect to irregularities are shown in Figures 9–11. A LMM-ANOVA with irregularities, dimensions, and axes as the fixed effect variable and with subjects as the random effect factor showed that irregularity level has no significant effects on accuracy [*F*(2,6.08) = 0.21, *p* = 0.82], reaction time [*F*(2,10.92) = 0.32, *p* = 0.73], and confidence [*F*(2,12.49) = 0.94, *p* = 0.42]. No interaction between irregularity and other factors was found to be significant in all tests. In addition, a LMM-ANOVA with session order as the main factor did not find any significant effect on accuracy [*F*(1,36.65) = 0.23, *p* = 0.64], reaction time [*F*(1,3.98) = 4.08, *p* = 0.11], or confidence level [*F*(1,5.27) = 2.04, *p* = 0.21], which indicates a non-significant effect from learning experience on subjects’ performance.

Our results indicate that observers were able to judge the motion rigidity of hypercubes along with the horizontal displacement but behaved much poorer when the stimulus moved along with the other two axes. Considering the non-significant effect of irregularity of objects’ shape on subjects’ performance, we hypothesized that dynamical horizontal viewpoint rather than the shape is a key in observers’ detection of objects’ motion rigidity. To further test the idea, we conducted Experiment 2, in which we asked subjects to actively observe the stimuli via spontaneous viewpoint change by allowing head movements. We expected an increase in subjects’ performance compared to fixed headset condition.

#### 2.5.2. Experiment 2

##### 2.5.2.1. Reported measurements

The five subjects who participated in Experiment 2 were identical to the five subjects who gave an acceptable performance in Experiment 1, and their percent correct performance herein were 100, 91.67, 99.07, 91.67, and 89.81%, respectively.

Figure 6 shows the accuracy of the dimensions and displacement axis. For the active headset condition, LMM-ANOVA, with displacement axes and dimensions as fixed effect variables and with subjects as the random effects grouping factor, was conducted but no effect was found to be significant on accuracy [Axis: *F*(1,5.33) = 0.38, *p* = 0.56; Dimension: *F*(1,4.7) = 5.92, *p* = 0.06; Axis × Dimension: *F*(1,4.1) = 0.36, *p* = 0.58]. Figure 7 shows the reaction time by the dimensions and displacement axes. Similarly, the LMM-ANOVA detected no significant effect from the displacement axis on reaction time [*F*(1,5.76) = 0.19, *p* = 0.68] nor from the interaction between axis and dimension [*F*(1,4.97) = 0.54, *p* = 0.5], but a significant effect of dimension [*F*(1,5.3) = 6.87, *p* = 0.04]. Moreover, as shown in Figure 8, given the results in accuracy and reaction time, the same model did not detect any significant effect on confidence level neither [Axis: *F*(1,10.12) = 0.61, *p* = 0.45; Dimension: *F*(1,4.02) = 3.31, *p* = 0.14; Axis×Dimension: *F*(1,5.26) = 0, *p* = 0.98]. Detailed measurements can be found in Table 2.

**Table 2:** Active headset condition: 3D measurement, 4D measurement in means (std).

	z-axis displacement	y-axis displacement
Accuracy (%)	97.04 (16.98), 89.21 (31.09)	96.59 (18.21), 92.27 (26.77)
Reaction Time (ms)	543.97 (289.04), 590.42 (294.07)	534.33 (298.23), 627.32 (318.93)
Confidence	4.83 (0.65), 4.32 (1.12)	4.84 (0.53), 4.32 (1.2)

Measurements with respect to irregularities are shown in Figures 9–11. A LMM-ANOVA with irregularity levels, dimensions, and axes as main factors showed that irregularity level had no significant effects on accuracy [*F*(2,9.26) = 0.9, *p* = 0.44], reaction time [*F*(2,4.64) = 0.78, *p* = 0.51], and confidence [*F*(2,5.45) = 0.14, *p* = 0.87]. No interaction between irregularity and other factors was found to be significant in all tests. In addition, a LMM-ANOVA with session order as the main factor did not find any significant effect on accuracy [*F*(1,4.29) = 3.5, *p* = 0.13] or reaction time [*F*(1,4.1) = 6.68, *p* = 0.06], but a significant effect on confidence level [*F*(1,33.36) = 8.71, *p* < 0.01]. These results suggest that subjects’ performance did not improve effectively from the learning experience. The mean (std) confidence was found to change from 4.5 (1.04) to 4.66 (0.86), raising a slight but significant increase.

##### 2.5.2.2. Comparisons between fixed and active headset conditions

As explained, preliminary data showed that active exploration would improve the performance in this rigidity motion discrimination task. Considering the ceiling accuracy observed in Experiment 1 under the x-axis displacement condition, in Experiment 2 we tested under the y-axis and z-axis conditions only. Therefore, to test the improvement in performance raised by the active headset statistically, here we first tested the effect of observation conditions (fixed headset and active headset) under the y-axis and z-axis conditions only. LMM-ANOVA with observation conditions and dimensions as fixed effect factors and with subjects as the random effect factor found that the observation condition had significant effects on all the three reported measurements [Accuracy: *F*(1,4.79) = 114.42, *p* < 0.001; Reaction time: *F*(1,4.01) = 17.52, *p* = 0.01; Confidence level: *F*(1,4.02) = 33.67, *p* < 0.01]. Referring to the values shown in Tables 1, 2, significant improvements took place in accuracy, reaction time, and confidence. In addition, compensated by these improvements, these performances can be comparable to how subjects behaved in Experiment 1 under the x-axis condition.

##### 2.5.2.3. Correlations between confidence levels and other performance measurements

**Figure 12:**
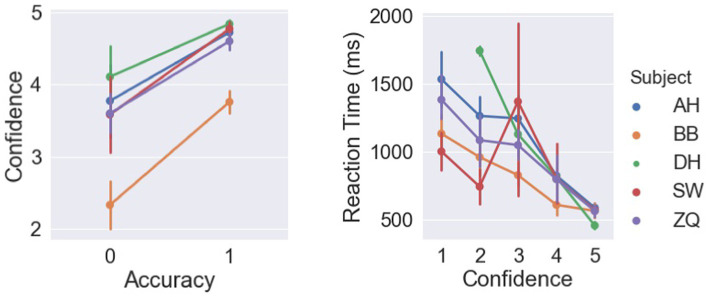
Left: Relations between confidence levels and accuracy. Right: Relations between reaction times and confidence.

In this study, we asked subjects to report their confidence levels based on a 1–5 scale to measure their cognitive access in detecting the rigid motion quantitatively. We expected that confidence levels are correlated to both reaction times and accuracy if subjects are aware of their performance during the experiments. As shown in Figure 12, higher confidence levels were reported in the correct trials compared to the incorrect ones, as a repeated-measure ANOVA found significant effect of accuracy on confidence [*F*(1,4) = 20.12, *p* = 0.01]. Similarly, a significant negative correlation was observed between the confidence levels and reaction times with a Pearson correlation test [*r* = −0.52, *p* < 0.001].

##### 2.5.2.4. Head movements during subjects’ active explorations

**Figure 13:**
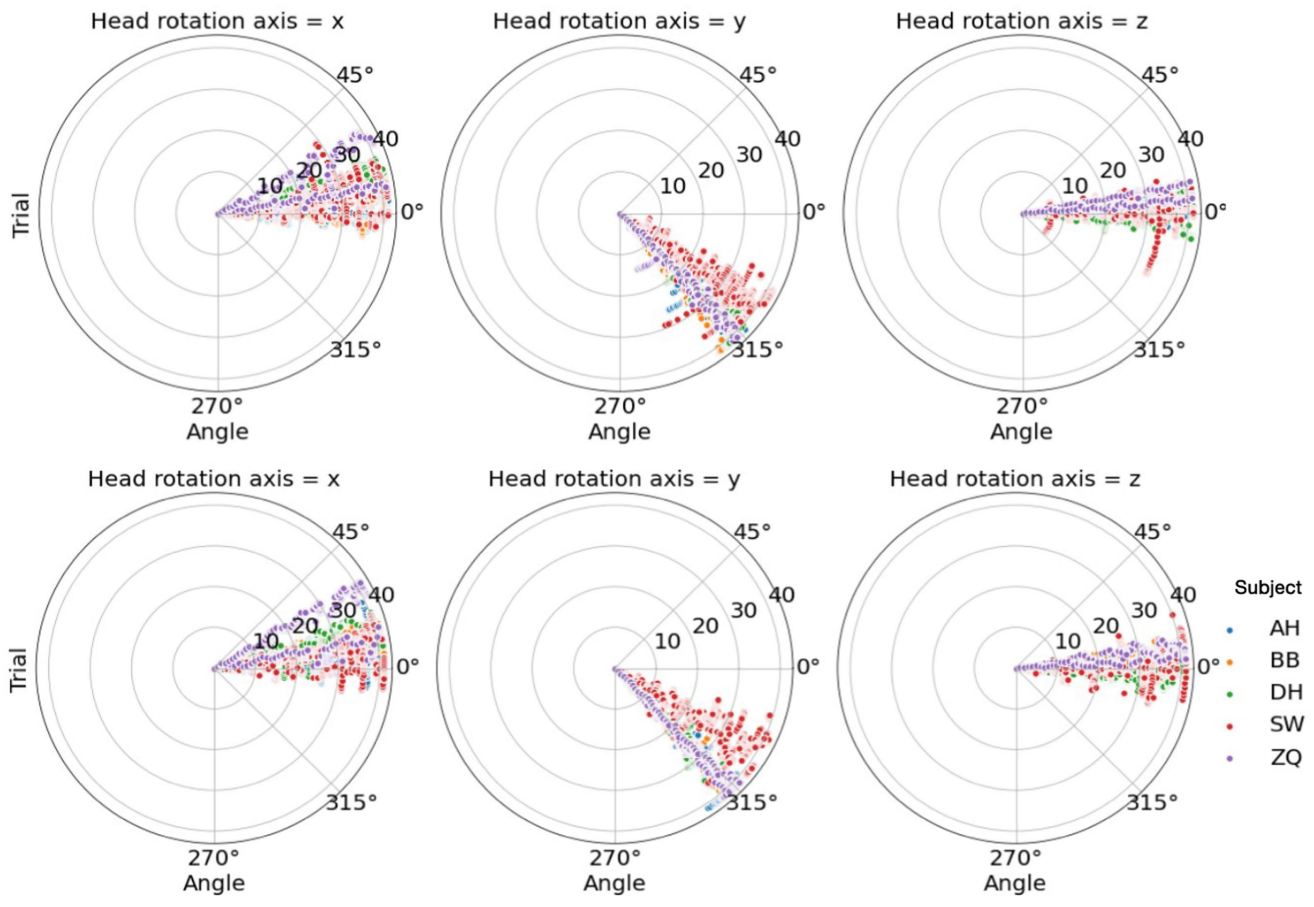
Top: Head rotation angles along each axis for each subject in the 3D condition. Bottom: Head rotation angles along each axis for each subject in the 4D condition.

To analyze the correlation between the head movement and the performance, we recorded the visual angles along the x, y, and z axes calculated by the angles between the headset’s viewing direction and the vector from the headset’s position to the cube’s position. In each trial, angles were scanned by approximately 18 Hz, and therefore about 54 data points were recorded during the 3-s observation period in each trial. We then calculated the median and the percentile range of angles collected along each axis in each trial to investigate the effect of viewpoint and its change on performance. The percentile ranges were calculated by the third quarter value subtracted from the first quarter value. We did not use the maximum and minimum as they are sensitive to outliers. As shown in Figure 13, we found that, for all subjects, the ranges of viewpoints within trials were mostly small and they were narrow across all trials. Besides, we did not find effective correlations between the range of viewpoints and the performance, as detailed in Table 3.

**Table 3:** Correlations between the headset viewpoint and the performance: Pearson correlation, value of *p*.

		3D			4D		
		Accuracy (%)	Reaction Time (ms)	Confidence	Accuracy (%)	Reaction Time (ms)	Confidence
Range	x	0.14, <0.01	0.18, <0.01	−0.09, 0.05	0.06, 0.2	0.19, <0.01	−0.1, 0.04
	y	0.1, 0.03	0.1, 0.03	−0.1, 0.03	0.04, 0.41	0.1, 0.01	−0.11, 0.02
	z	0.09, 0.05	0.04, 0.37	0.01, 0.86	0.04, 0.37	0.02, 0.62	0.03, 0.6
Median	x	0.15, <0.01	−0.13, <0.01	0.07, 0.14	−0.06, 0.26	0.2, <0.01	−0.11, 0.03
	y	−0.29, <0.01	0.02, 0.72	−0.07, 0.17	−0.18, <0.01	0.02, 0.74	−0.09, 0.05
	z	0.06, 0.2	0.06, 0.22	−0.05, 0.29	−0.13, <0.01	−0.01, 0.84	−0.08, 0.11

As for the median of the angles, we found subjects tended to have their own preferred viewpoints. These viewpoints varied along the x-axis across subjects and spread narrowly along the z-axis within 15 deg., which can be resulted from different heights across subjects and people’s usual horizontal head statics. Interestingly, these angles were generally around 45 deg. along the y-axis, which implied a common optimal observation viewpoint selected by the subjects, in spite that there was no strong correlation between the viewpoint and the performance. In fact, the active headset remarkably improved the performance compared to the fixed headset condition and strong ceiling effects were observed. This ceiling performance could weaken its correlation to the headset movement.

## 3. Discussion

Our results show that, given 3D spatial cues, subjects can learn 4D object motion and respond as fast and accurately as for 3D stimulus. This is shown by no significant difference in accuracy and reaction time between cubes and hypercubes.

With the headset fixed and objects’ vertices displaced along the x-axis, accuracies in both 3D and 4D recognition are close to perfect and therefore a ceiling effect might exist and reduce the difference resulting by the dimensionality. However, by changing the displacement axis to increase the difficulty, all dependent variables change equally between 3D and 4D conditions, showing a similar dependence on 3D information in both 3D and 4D object motion recognition tasks. Additional support comes from the finding of a compensatory effect on the performance from active exploration or variable viewpoints in both 3D and 4D conditions. With the active headset, subjects’ performance in recognizing objects displaced along difficult axes became as well as the easy axis. Surprisingly, this compensatory effect can be triggered with very slight head movements and viewpoint changes, even in 4D tasks. Consistent to our expectation, subjects reported confidence levels that were significantly higher in correct trials compared to incorrect trials, and their confidence was negatively correlated to the reaction times. These findings suggest that subjects were able to generate phenomenal experience based on the 4D stimuli we presented. Importantly, in our data we did not find a significant effect from experience as performance on two sessions were similar, and therefore, concluded that participants may simply use available neural machinery to detect and discriminate relevant visual cues to perform the 4D task.

Despite the fact that the x-axis and y-axis shared the same front viewpoint toward the stimuli, subjects showed superiority in performance along the x-axis in the fixed headset condition. This might be due to the fact that our stimuli were shown according to a vertical arrangement, in which one object was shown on the top whereas another one was shown at the bottom. Another possible reason is that displacement along the x-axis causes the vertical orientation change, which has been suggested to convey more binocular disparity information for the stereopsis system (Yazdanbakhsh and Watanabe, 2004). A second notable axis effect was the asymmetry of accuracies in 3D and 4D tasks between the y-axis and z-axis shear conditions, as shown in Figure 6. One possible explanation is the difference between 3D motion and 4D motion. The 3D motion in our experiments was rotation along a single axis. However, the 4D motion was rotation along a plane defined by two axes and this motion was then projected back to 3D to be presented. Therefore, the 4D motion in each axis condition actually consisted of displacements along all three axes with a matter of extent. This might produce interactive effects between different axis displacements and cause improved or deteriorated performance compared to the 3D conditions.

In addition to increasing the difficulty of tasks at a spatial-dimensional level, an object structural-level factor, irregularity, was also studied. However, it was found to have no effect on the subjects’ performance in these tasks. This implies that subjects’ motion processing in both 3D and 4D tasks was not from shape analysis but spatial speculations. This finding shows that subjects were not recognizing hypercubes using pure 3D cues without hyperdimensional constructions, since 3D cues are shape-level information for the 4D objects. In spite of extracting spatial information beyond 3D, materialized as rigidity of projected hypercube within the 3D setup experiments, 4D phenomenal experience is not there yet. Therefore, we interpret our findings in this study as “3 $\frac{1}{2}$ D perception.”

In summary, our results add to the corpus of findings showing humans’ ability to perceive higher-dimensional visual stimuli in a broad sense. In terms of phenomenology, one can compare the 4D percepts induced thus far to those of 3D percepts emerging from 2D stimuli: For example, by using perspective cues, we can “perceive” 3D structure from 2D drawings. That percept is not as rich as one that we would get in a VR set generating 3D percepts using disparity and motion cues. What remains to be seen is whether one can generate 4D percepts similar to 3D percepts in a VR by using extensive training.

## Data availability statement

The raw data supporting the conclusions of this article will be made available by the authors, without undue reservation.

## Ethics statement

The studies involving humans were approved by the University of Denver Institutional Review Board for the Protection of Human Subjects. The studies were conducted in accordance with the local legislation and institutional requirements. The participants provided their written informed consent to participate in this study.

## Author contributions

DH collected data by conducting the experiments, performed the analyses, and prepared the manuscript. D-TN has developed all the experimental programs using the virtual reality setup. HO contributed to the elaboration of the experimental design and conceptualization of the overall study and to the brushing up of the manuscript. SN proposed the 4D rigidity task and the basic experimental design. AY contributed to the overall facilitation of the project and to the conceptualization of the study and refinement of the manuscript. All authors contributed to the article and approved the submitted version.

## Conflict of interest

SN was employed by Honda Research Institute Japan Co., Ltd.

The remaining authors declare that the research was conducted in the absence of any commercial or financial relationships that could be construed as a potential conflict of interest.

## Publisher’s note

All claims expressed in this article are solely those of the authors and do not necessarily represent those of their affiliated organizations, or those of the publisher, the editors and the reviewers. Any product that may be evaluated in this article, or claim that may be made by its manufacturer, is not guaranteed or endorsed by the publisher.
