# Supplementary material for: Perception of rigidity in three- and four-dimensional spaces
Source: Front Psychol. 2023 Aug 17;14:1180561. doi: 10.3389/fpsyg.2023.1180561 (PMC10470465; doi:10.3389/fpsyg.2023.1180561)
Supplement: Supplementary file 1 [file Presentation_1.pdf]

Mathematical descriptions of the stimuli

The stimuli used in this study included the 3D cubes and 4D tesseracts in different levels of irregularity that were constructed by vertices and edges connecting them. In the following description, we use the vector, c_k, to indicate the coordinate of a cube or a tesseract's vertex k (cube: $k = \{1,2, \dots ,8\}$; tesseract: $k = \{1,2, \dots ,16\}$) and thus define the indexes 0, 1, and 2 that correspond to the x, y, and z axes respectively. For the tesseract, an additional axis, w, was added, which corresponds to index 3 in all the vectors and matrixes as follows. Thus, all the vectors in 3D space have three elements, whereas they have four elements in 4D space.

Here we define two basic matrix transformations used in this section: rotation and shear in Equations 1 and 2 respectively. For an n-dimensional object, these matrixes are $n \times n$. Let $R_{a,b}(\theta)$ to indicate the coordinate change due to the positive rotation from axis b to axis a relative to the origin for θ.

$$R_{a,b}(\theta) = \left(r_{i,j} \middle| \begin{array}{c} r_{a,a} = cos\ (\theta) \\ r_{b,b} = cos\ (\theta) \\ r_{a,b} = -sin\ (\theta) \\ r_{b,a} = sin\ (\theta) \\ r_{j,j} = 1, j \neq aorb \\ r_{i,j} = 0, elsewhere \end{array} \right) \quad (1)$$

It can be easily verified that $R_{x,y}$, $R_{y,z}$, and $R_{z,x}$ respectively indicate to positive rotation around z, x, and y axes in the 3D space. Extended to 4D space, $R_{x,w}$, $R_{y,w}$, and $R_{z,w}$ respectively correspond to the positive rotation around y-z, x-z, and x-y planes.

Also, axial displacement via shearing was used in the non-rigid motion. For example, as illustrated in Figures 3 and 4, by shearing y-axis to x-axis, left-right (x-axis) displacement is created. Similarly, in-out (z-axis) displacement and up-down (y-axis) displacement were created by shearing y-axis to z-axis and shearing z-axis to y-axis respectively. The transformation matrix shearing axis b to axis a with a degree of σ can be determined by Equation 2.

$$S_{a,b}(\sigma) = \left(s_{i,j} \middle| \begin{array}{c} s_{a,b} = \sigma \\ s_{j,j} = 1 \\ s_{i,j} = 0, elsewhere \end{array} \right) \quad (2)$$

1. 3D Rigid Motion

The rigid motion of the 3D cubes was practically realized by adding the random vertex movements and the rotation along x and y axes. The random movements for the vertex k, u_k, was a vector whose three elements were independently generated from a uniform distribution $U(-0.1,0.1)$.

Therefore, during rigid motion of a 3D cube, the vertex k's movement with respect to time, $v_k^{3r}(t)$, can be determined by the Equation 3.

$$v_k^{3r}(t) = R_{z,y}(\theta_1(t)) \cdot R_{x,z}(\theta_2(t)) \cdot (c_k + u_k) \tag{3}$$

where $\theta_1(t)$ and $\theta_2(t)$ are smooth random time series generated with two independent Gaussian processes that are subject to $N(0,1)$.

2. 3D Non-Rigid Motion

The non-rigidity of the stimuli's motion was created by firstly deforming the shape of the object, which was achieved by adding random positional shifts, f_k, to the vertices of stimuli, as indicated by Equation 4. This random shift was synchronized with the rotation around the y-axis to avoid creating obvious discrimination cues.

$$f_k(t) = \mu\theta_2(t)e_k \tag{4}$$

where μ is a constant indicating the degree of synchronization, and e_k is a unit vector whose direction is uniformly distributed across the entire 3D space.

After the deformation, an axial displacement was added via shearing one axis to another. Importantly, the degree of shearing in the stimuli was also synchronized with the rotation around the y-axis. Therefore, the vertex k's movement with respect to time during non-rigid motion that shears axis b to axis a can be represented by Equation 5.

$$v_k^{3n}(t) = R_{z,y}(\theta_1(t)) \cdot R_{x,z}(\theta_2(t)) \cdot S_{a,b}(\theta_2(t)) \cdot (c_k + u_k + f_k(t)) \tag{5}$$

3. 4D Motion

Similar to the 3D rigid motion, the 4D tesseracts' rigid motion were realized by adding the random vertex movements and the rotation along y-z, x-z, and x-y planes. Therefore, the vertex k's movement with respect to time during rigid motion, $v_k^{4r}(t)$, can be determined by the Equation 6.

$$v_k^{4r}(t) = R_{x,w}(\theta_1(t)) \cdot R_{y,w}(\theta_2(t)) \cdot R_{z,w}(\theta_3(t)) \cdot (c_k + u_k) \tag{6}$$

Moreover, the vertex k's movement with respect to time during non-rigid motion that shears axis b to axis a can be represented by Equation 7.

$$v_k^{4n}(t) = R_{x,w}(\theta_1(t)) \cdot R_{y,w}(\theta_2(t)) \cdot R_{z,w}(\theta_3(t)) \cdot S_{a,b}(\theta_2(t)) \cdot (c_k + u_k + f_k(t)) \tag{7}$$
